# Pupal productivity of larval habitats of *Aedes aegypti* in Msambweni, Kwale County, Kenya

**DOI:** 10.1007/s00436-022-07777-0

**Published:** 2023-01-23

**Authors:** Alawih S. Mwakutwaa, Harun N. Ngugi, Bryson A. Ndenga, Amy Krystosik, Moses Ngari, Laila U. Abubakar, Shadrack Yonge, Uriel Kitron, A. Desiree LaBeaud, Francis M. Mutuku

**Affiliations:** 1 Department of Environment and Health Sciences, Technical University of Mombasa, Mombasa, Kenya; 2 Department of Biological Sciences, Chuka University, Chuka, Kenya; 3 Centre for Global Health Research, Kenya Medical Research Institute, Kisumu, Kenya; 4 Department of Pediatrics, Division of Infectious Diseases, Stanford University School of Medicine, Stanford, CA USA; 5 KEMRI-Wellcome Trust Research Programme, Kilifi, Kenya; 6 Department of Pure and Applied Sciences, Technical University of Mombasa, Mombasa, Kenya; 7 Department of Environmental Sciences, Emory University, Atlanta, GA USA

**Keywords:** *Aedes aegypti*, Pupal productivity, Dengue virus, Zika virus, Chikungunya virus

## Abstract

*Aedes aegypti* is an important vector of several arboviruses including dengue and chikungunya viruses. Accurate identification of larval habitats of *Ae. aegypti* is considered an essential step in targeted control. This study determined *Ae. aegypti* productivity in selected larval habitats in Msambweni, Kwale County, Kenya. Three sequential larval habitat surveys were conducted. The first survey was habitat census (baseline) through which 83 representative larval habitats were identified and selected. The second and third surveys involved estimating daily productivity of the 83 selected larval habitats for 30 consecutive days during a wet and a dry season, respectively. Of 664 larval habitats examined at baseline, 144 larval habitats (21.7%) were found to be infested with *Ae. aegypti* larvae. At baseline, majority (71%) of the pupae were collected from two (2/6) larval habitat types, tires and pots. Multivariate analysis identified habitat type and the habitat being movable as the predictors for pupal abundance. During the 30-day daily pupal production surveys, only a few of the habitats harbored pupae persistently. Pupae were found in 28% and 12% of the larval habitats during the wet and dry seasons, respectively. In the wet season, drums, tires, and pots were identified as the key habitat types accounting for 85% of all pupae sampled. Three habitats (all drums) accounted for 80% of all the pupae collected in the dry season. Predictors for pupal productivity in the wet season were habitat type, place (whether the habitat is located at the back or front of the house), habitat purpose (use of the water in the habitat), and source of water. Although the multivariate model for habitat type did not converge, habitat type and habitat size were the only significant predictors during the dry season. Drums, pots, and tires were sources of more than 85% of *Ae. aegypti* pupae, reinforcing the “key container concept.” Targeting these three types of habitats makes epidemiological sense, especially during the dry season.

**Supplementary Information:**

The online version contains supplementary material available at 10.1007/s00436-022-07777-0.

## Background

Accurate identification of epidemiologically important types of larval habitats is considered an essential step in targeted control of *Ae. Aegypti*, an important vector for several arboviruses including dengue and chikungunya viruses. *Stegomyia* indices that have traditionally been used for routine surveillance that assume abundance of *Aedes* vector mosquitoes is antecedently proportional to the risk of *Aedes-*borne diseases. However, a high rate of mosquito immatures’ mortality makes *Stegomyia* indices less sensitive in identification of the “key” larval habitats for *Ae. Aegypti* (Barrera et al. 2006a; Focks and Chadee 1997; Hammond et al. 2007; Tun-Lin et al. 2009; Wijayanti et al. 2016). Quantifying pupal productivity in the larval habitats has emerged as the best alternative, because pupae are a better proxy for adult female mosquitoes (Barrera et al. 2006b; Focks and Alexander 2006; Focks et al. 2000; Wijayanti et al. 2016).

Productivity of *Ae. aegypti* larval habitats is influenced by several factors, including location, water purpose, cover, frequency of use, shade, movement, water volume, containers’ exposed surface, and frequency of refilling and emptying (Barrera et al. 2006b; Philbert and Ijumba 2013; Troyo et al. 2008; Vezzani and Albicocco 2009). Containers used for water storage keep water for periods long enough for complete larval development, and hence tend to contain a higher density of larvae and pupae than containers used for drinking water. This is also the case for the containers that are frequently used (Forsyth et al. 2020; Midega et al. 2006). Containers holding larger volumes of water such drums and buckets were found to contain large amount of pupae compared to containers with a small amount of water (Lenhart et al. 2006). More breeding activity is observed in containers that are uncovered than those that are tightly covered (Maciel-de-Freitas et al. 2007; Morrison et al. 2006). Depending on geographical setting, containers vary in productivity based on their location. In some studies, indoor containers have been found to be more productive (Barrera et al. 2006a; Midega et al. 2006). Other studies have reported more outdoor breeding (Islam et al. 2019; Lutomiah et al. 2016; Ngugi et al. 2017; Saifur et al. 2012; Wongkoon et al. 2007). Key containers can be determined by the number of pupae in a particular container; therefore, productivity of containers can be estimated best by counting of pupae, as opposed to counting the number of larvae. This is because the number of pupae tends to correlate more with that of adult mosquitoes (Chadee et al. 2009; Midega et al. 2006). Compared to larvae, pupae mortality rate is low, they are easy to count, and the length of time from pupae to adult is short (Bisset et al. 2006; Focks and Chadee 1997).

The aim of this study was to determine pupal productivity of larval habitats for *Ae aegypti* and the factors associated with productivity in Msambweni, Kwale County in south coast of Kenya. Determination of habitat-specific productivity provides a fairly accurate estimate of entomological risk for an area-wide plan. Knowledge of productivity of different container types is vital for targeted control of *Ae aegypti*, and thus can contribute to the reduction of dengue transmission in the region.

## Materials and methods

### Study area

The survey was conducted in a 600-m^2^ transect at Bomani town, Msambweni location, Kwale County, Kenya. Bomani is an upcoming urban center with a population density of 958–km^2^ (Kenya National Bureau of Statistics, 2019). The site is located 60 km south of Mombasa city. The coastal climate is tropical hot and humid throughout the year with annual temperature of 23 to 34 °C and average relative humidity of 60 to 80%. The study area is approximately 2 km from the Indian Ocean seashore and very close to the main hospital in Kwale County: Msambweni County Referral Hospital. The main activities of the people in the area are small-scale farming and fishing. Residents in the study site store water for domestic use in various containers because tap water supply is not reliable. Water supply is mostly from wells, boreholes, and harvested rainwater that supplement inadequate pipe water supply.

### Study surveys

This study was conducted through three sequential surveys. The first survey is herein referred to as the “larval habitat census” or “baseline survey” involved identifying the study area and identifying all the potential larval habitats. Additionally, larvae and pupae abundance was estimated as well as collecting information on container management practices and characteristics. This was followed by the “wet season survey.” Daily pupal productivity was estimated for 30 consecutive days in 83 selected representative larval habitats during a wet season. The “dry season survey” was conducted during a dry season. This survey involved estimating daily pupal productivity for 30 consecutive days in 83 (same as in the wet season survey) selected representative larval habitats.

### Larval habitat census

A larval habitat census was conducted from June 2 to June 17, 2017, to map and document all water receptacles within a 600 by 600 m area in Bomani town, Msambweni location, Kwale County. Potential larval habitats in outdoor domestic environment of every house located within the selected area were inspected for mosquito larvae and pupae. A water receptable found harboring any of the four mosquito larval instars and/or pupae were considered as a positive larval habitat. The larval habitats were classified into different habitat types as described by Ngugi and others (Ngugi et al. 2017). All pupae and a sample of larvae (third and fourth instars) from positive larval habitats were collected with the aid of pipettes and ladles (Chadee et al. 2007), counted, and recorded on field-data forms. Water from large larval habitats was first sieved, and mosquito samples were placed in a white plastic tray with some water from which the immatures were pipetted. Other than the small volumes of water taken away with mosquito samples, the rest was retained in both small and large larval habitats. Mosquito samples were placed in 10-ml falcon tubes and/or Whirl–pak® plastic bags (Nasco, Fort Atkinson, WI), labeled, and taken to the Vector Borne Disease Control Unit (VBDCU) laboratory at Msambweni County Referral Hospital. Immature mosquitoes were reared in 200-ml plastic cups under laboratory conditions at an average temperature of 28.15 ± 1.8 °C and relative humidity of 80.9 ± 6.3%, and larvae were fed on TetraMinbaby® fish food (Melle, Germany). Emerged adults were identified to species using standard taxonomic keys (1941). *Ae*. *aegypti* (L) subspecies were morphologically distinguished using keys by Edwards (1941), Mattingly (1958), and Huang (2004).

### Container management practices and characteristics

A total of seven container types were identified and classified based on their use and material: drums, tires, pots, small domestic containers (SDC), buckets, jerrycans, and others (Ngugi et al. 2017). Drums were defined as 100–500-l capacity plastic or metal water storage containers. Pots included flower vases and water storage vessels made of clay. Small domestic containers included small plastic food containers, tins, bottles, plates, cans, cooking pots (sufuria), and jars. Others included polythene bags, fallen leaves, coconut shells, hoof prints, drains, gutters, septic tanks, shoes, cisterns, sinks, and animal feeding containers (AFCs). The AFCs, ranged from small 1-l bird watering and feeding containers made of plastic or cut tires, to a medium 30-l plastic container for watering cattle. For each breeding habitat, data was collected on the location within the outdoor domestic environment (frontyard, backyard, and others including bushes, gardens, dumpsites), container size or capacity (small < 25 l; large > 25 l), capable of being moved (movable; not movable), exposure to sunlight (fully shaded from sunlight; partially shaded from sunlight; fully exposed to sunlight), purpose of the water in the water storage containers (domestic uses; no purpose), evidence of covering (covered; not covered), water source, and frequency of water refilling.

### Estimation of *Ae. aegypti* larval habitat productivity

A total of 83 representative habitats were randomly selected from the 664 potential larval habitats identified during the habitat census and marked with indelible ink for ease of identification. Daily productivity was estimated in the 83 selected representative larval habitats for 30 consecutive days (wet season: July 14 to August 12, 2017; dry season: February 28 to March 29, 2018). The selected larval habitats included 17 buckets, 9 drums, 9 jerrycans, 5 others, 8 pots, 16 SDCs, and 19 tires. At every habitat daily for 30 days during the two sampling periods, quantification of the numbers of *Ae. aegypti* immatures was done following methods as described by Chadee and others and Ngugi and others (Chadee et al. 2007; Ngugi et al. 2017). The number of larvae and their stages of development were recorded for each habitat as well as the number of pupae. All pupae were removed and allowed to emerge in the laboratory. All emerged adults were identified to sub-species level by morphological features (Edwards 1941). During each sampling visit, records were made on the location within the outdoor domestic environment (frontyard, backyard, and others including bushes, gardens, dumpsites), container size or capacity (small < 25 l; large > 25 l), capable of being moved (movable; not movable), exposure to sunlight (fully shaded from sunlight; partially shaded from sunlight or fullly exposed to sunlight), purposes of the water in the water storage containers (domestic uses; no purposes), evidence of covering (covered; not covered), water source, and frequency of water refilling or emptied.

### Data analysis

Data analysis was conducted independently for the 3 datasets in this study: the baseline survey and the wet and dry season longitudinal datasets. Descriptive analyses were used to explore the data. The number of pupae per habitat was the output variable for the baseline survey and for both the wet and dry season longitudinal surveys. Pearson dispersion statistic was used to assess for over-dispersion for all the 3 datasets. All three datasets were highly over-dispersed with 86% zero counts in the baseline survey and over 90% zero counts in both the wet and dry season surveys. A zero-inflated negative binominal regression (ZINB) was therefore considered appropriate to test association with *Ae. aegypti* pupae infestation (Heilbron 1994). The ZINB model fits a negative binomial component and a zero-inflated component, since we were interested with the association with *Ae. aegypti* pupae infestation estimated by the negative binomial component, only its regression coefficients transformed into incidence rate ratios (IRRs) were reported. Before being included in the ZINB models, correlations among the predictors were assessed using Spearman’s rank correlation coefficient and those with strong correction (> 0.7) excluded in the analysis. For the baseline survey, factors assessed for association with risk of *Ae. aegypti* pupae infestation included larval habitat type (abbreviated as “habitat type”), location within the outdoor domestic environment (abbreviated as “place”), container size, capable of being moved (abbreviated as “move”), exposure to sunlight (abbreviated as “shade”), purpose of the water in the water storage containers (abbreviated as “water purpose”), evidence of covering (abbreviated as “cover”), water source, and frequency of water refilling (abbreviated as “filled”). All these factors were included in univariate ZINB regression models, and those with *P* values < 0.1 were included in the multivariate ZINB regression model.

For the wet and dry season datasets, we assessed the daily pupal productivity of the larval habitats by running both univariate and multivariate ZINB models controlling for the multiple daily observations using robust standard errors. A similar approach to the one used in baseline survey above was used to fit multivariate ZINB models for wet and dry seasons. The pupal productivity predictors were habitat type, place, container size, shade, water purpose, cover, habitat filled, water source, and habitat stability. The predictor “move” was not included in this analysis because no pupae were recorded from the unmovable larval habitats. Habitat stability was defined as the number of days a habitat contained water during the 30-day sampling period. Habitats were classified as stable if they had water for at least 7 days in wet season and 20 days for the dry season. The habitat stability cutoffs were selected to maximize the number of habitats within each category and varied among the wet and dry seasons. Statistical analyses were carried out using SAS software version 9.3 (SAS Institute Inc., Cary, NC, USA) and Stata version 15.1 (StataCorp, College Station, TX, USA).

## Results

### Habitat census

All 211 houses located within the 600 × 600 m^2^ area of Bomani town were visited during the larval habitat census from June 2 to 17, 2017. Potential larval habitats were found in the outdoor domestic environment of 82% (172) of the houses. A total of 664 potential larval habitats were identified and classified based on their use and material into seven habitat types including buckets, drums, jerrycans, pots, small domestic containers (SDCs), tires, and others (Table 1). Only a quarter of the habitats (169) with water were positive for mosquitoes. Of the 169 positive habitats, 83%, 9%, and 6% were inhabited by *Ae. aegypti*, culicine, and toxorhynchites immatures, respectively. Two percent of the positive habitats had both *Ae. aegypti* and culicine immatures. Tires were the preferred habitats by the three different mosquito species found in the inspected containers. Buckets (35%) were the most abundant habitat type (Table 1). The number and different types of larval habitats identified during larval habitat census and the mosquito species found in them are shown in Table 1.

**Table 1:** Types of larval habitats identified during the baseline survey

Habitat type	No. of habitats with *Ae. aegypti* larvae only	No. of habitats with culicine larvae only	No. of habitats with Toxorhynchites larvae only	No. of habitats with *Ae. aegypti* and culicine larvae	No. of habitats without larvae	Total, *N* (%)
Bucket	28	1	0	1	205	**235 (35.4)**
Drum	11	2	0	1	31	**45 (6.8)**
Jerrycans	21	0	1	0	97	**119 (17.9)**
Pots	9	1	0	0	12	**22 (3.3)**
SDC	16	0	0	0	114	**130 (19.6)**
Tire	53	11	9	2	17	**92 (12.3)**
Other	4	0	0	0	17	**21 (4.7)**
Total, *N* (%)	**140 (21.1)**	**15 (2.3)**	**10 (1.5)**	**4 (0.6)**	**495 (74.5)**	**664 (100.0)**

The results presented are counts, apart from totals that are *n* (%)

### *Ae. aegypti* abundance

Of 664 larval habitats examined, 144 larval habitats (21.7%) were found to be infested with *Aedes aegypti* larvae. The mean number of wet containers per house was 9.28 (SD = 8.60, median = 7). In total, 6162 and 1097 larvae and pupae, respectively, were collected from the 172 houses. The summary statistics for larval and pupal abundance during the baseline are provided in Table 2. Containers with higher mosquito abundance were not the most common containers for pupae; tires which represented 14% of the total habitats and with infestation rate of 59.8% were found harboring 60% of the pupae. Seventy-one percent of the pupae were collected from tires and pots combined, which together represented 17% of the habitats. On the other hand, buckets and SDC which represented 55% of the total habitats had an infestation rate of 11.8%, yet only 13.5% of the pupae was found in them (Table 2). The results of both univariate and multivariate analysis of predictors for *Ae. aegypti* pupal abundance are presented in Table 3. Multivariate analysis showed that only habitat type and the habitat being movable were associated with pupal abundance (Table 3).

**Table 2:** Summary statistics for larval and pupal productivity during the baseline survey

Habitat type	No. of larval habitats (+ ve)	Percentage of larval habitats + ve	No. of larvae	No. of pupae	Mean larvae/larval habitat ± SE	Mean pupae/larval habitat ± SE
Habitat type						
Buckets	235 (28)	11.9	736	70	3.1 ± 18.2	0.3 ± 1.6
Drums	45 (12)	26.7	1961	57	45.6 ± 160.7	1.3 ± 4.0
Jerrycans	119 (21)	17.6	436	95	3.7 ± 12.6	0.8 ± 4.8
Pots	22 (9)	40.9	653	125	29.7 ± 67.5	5.7 ± 11.0
SDC*	130 (15)	11.5	569	78	4.4 ± 31.7	0.6 ± 3.0
Others	21 (4)	19.0	31	14	1.8 ± 3.8	0.7 ± 2.6
Tires	92 (55)	59.8	1776	658	19.3 ± 40.9	7.2 ± 15.9
Water purpose						
No purpose	260 (74)	28.5	11,053	862	42.5 ± 132.1	3.3 ± 9.9
Domestic chores	404 (20)	5.0	3169	235	7.8 ± 50.0	0.6 ± 4.4
Container size						
Small	321 (67)	20.9	7899	810	24.6 ± 74.8	2.5 ± 8.4
Large	343 (27)	7.9	6323	287	18.4 ± 107.0	0.8 ± 5.8
Habitat filled						
≥ Week	203 (80)	39.4	12,473	981	61.4 ± 150.5	4.8 ± 12.2
≤ Week	461 (14)	3.0	1749	116	3.8 ± 38.2	0.3 ± 1.9
Place						
Front	109 (24)	22.0	2385	345	21.9 ± 54.1	1.4 ± 6.2
Back	555 (70)	12.6	11,837	752	21.3 ± 98.7	3.2 ± 11.1
Water source						
Rain	439 (85)	19.4	12,358	984	28.2 ± 106.1	2.2 ± 8.1
Others	225 (9)	4.0	1864	113	8.3 ± 56.9	0.5 ± 5.0
Move						
Yes	632 (87)	13.8	12,487	1058	19.8 ± 85.4	1.7 ± 7.4
No	32 (7)	21.9	1735	39	54.2 ± 186.7	1.2 ± 3.1
Cover						
Covered	104 (10)	9.6	1251	86	12.0 ± 48.0	0.8 ± 3.8
Uncovered	560 (84)	15.0	12,971	1011	23.2 ± 98.9	1.8 ± 7.7
Shade						
Sunlit	619 (90)	14.5	13,149	1058	21.2 ± 92.9	1.7 ± 7.4
Shaded	45 (4)	8.9	1073	39	23.8 ± 93.3	0.9 ± 4.0

*SE* standard error, *SDC* small domestic containers, + ve positive

**Table 3:** Univariate and multivariate analysis of predictors for *Ae. aegypti* pupal abundance

	Univariate analysis		Multivariate analysis	
	Crude IRR*	*P* value	Adjusted IRR*	*P* value
	(95% CI)		(95% CI)	
Habitat type				
Bucket	Reference		Reference	
Drum	1.76 (0.62, 5.04)	0.289	3.22 (0.93, 11.14)	0.06
Jerrycans	1.47 (0.62, 3.47)	0.380	1.47 (0.63, 3.43)	0.37
Other	1.3 (0.25, 6.62)	0.752	1.30 (0.26, 6.45)	0.75
Pots	2.58 (1.03, 6.45)	0.043	2.58 (1.05, 6.36)	0.04
SDC	1.45 (0.59–3.57)	0.421	1.45 (0.60, 3.52)	0.41
Tire	2.91 (1.47–5.76)	0.002	3.05 (1.55, 6.00)	0.00
Place				
Backyard	Reference		‡	
Frontyard	1.34 (0.80, 2.24)	0.267	‡	
Habitat size				
Large	Reference		‡	
Small	1.14 (0.69–1.88)	0.615	‡	
Move				
No	Reference		Reference	
Yes	2.18 (0.90, 5.28)	0.083	3.04 (1.07, 8.65)	0.037
Habitat purpose				
Domestic chores	Reference		‡	
No purpose	1.01 (0.58–1.75)	0.975	‡	
Habitat filled				
<Week	Reference		‡	
≥Week	1.48 (0.78–2.81)	0.230	‡	
Habitat shade				
Partial	Reference		‡	
Full shade	1.21 (0.39–3.73)	0.745	‡	
Cover				
No cover	Reference		‡	
Covered	0.71 (0.34–1.50)	0.373	‡	
Water source				
Others	Reference		‡	
Rain	0.92 (0.43–1.99)	0.836	‡	

*IRR* incidence rate ratio

^‡^Variables excluded in the multivariate regression model

^*^IRR and *P* values from the zero-inflated negative binomial regression models

### Wet and dry season pupal productivity

Table 4 summarizes the number of visits made to each habitat type and the total number of late larvae and pupae sampled during both wet and dry surveys. A total of 2340 visits to 83 habitats during a 30-day period of study for wet season survey was conducted. In the dry season survey, 1860 visits to 67 habitats were conducted. A total of 10,779 *Ae. aegypti* larvae and 2064 pupae was sampled during the wet season, and 2537 *Ae. aegypti* larvae and 365 pupae were sampled during the dry season (Table 4). Culicine larvae and pupae were rarely encountered and were not retained. All the reared *Aedes* mosquitoes were identified as *Ae. aegypti*. The habitat type “others” included small cooking pots, plastic bags, bottles, and coconut shells. None of the “others” habitats were found with larvae during both surveys. The “other” habitat type was therefore excluded from further analysis. The predictor “move” was not included in the univariate and multivariate analysis because no late instar larvae or pupae were found in the unmovable larval habitats during both wet and dry season surveys (Tables 4 and 5). In the dry season survey, small domestic containers (SDCs) were also excluded from the analysis because they were usually found without water or without *Ae. aegypti* immatures when found with water. “Habitat filled” was also not included in the multivariate ZINB models during the dry season because no pupae were found in the high-frequently water-refilled larval habitats (≥ week) category.

**Table 4:** Summary statistics for larval and pupal productivity during wet and dry season surveys

Variable	Wet season (Jul–Aug 2017)			Dry season (Feb–Mar 2018)		
	No. of visits	Total late instar larvae	Total pupae sampled	No. of visits	Total late instar larvae	Total pupae
Habitat type						
Buckets	510	60	56	510	12	3
Drums	270	4583	877	270	1845	248
Jerrycans	270	462	107	270	15	4
Pots	240	646	133	240	362	66
SDC	480	201	161	-	-	-
Tires	570	4827	730	570	303	44
Total	**2340**	**10,779**	**2064**	**1860**	**2537**	**365**
Habitat stability						
Unstable	1080	81	24	1410	387	62
Stable	1260	10,698	2040	450	2150	303
Place						
Backyard	330	1608	288	1620	2160	289
Frontyard	2010	9171	1776	240	2377	76
Habitat size						
Large	450	6925	1200	450	1860	258
Small	1890	3854	864	1410	677	107
Habitat purpose						
Domestic chores	1500	4889	1103	1020	1862	251
No purpose	840	5890	961	840	675	114
Habitat filled						
≤Week	1926	364	132	1599	97	0
≥Week	414	10,415	1932	261	2440	365
Habitat shade						
Partial shade	2040	9243	1825	1617	2518	355
Full shade	300	1536	239	243	19	10
Cover						
No cover	2114	9555	1996	1580	2479	352
Covered	226	1224	68	280	58	13
Water source						
Others	1163	2289	221	422	2389	359
Rain	1177	8490	1843	2068	148	6
Move						
No	492	0	0	249	0	0
Yes	1848	10,779	2064	1611	2537	365

**Table 5:** Mean number of larvae and pupae during wet and dry surveys in Bomani town, Kwale County, Kenya

	Wet season (Jul–Aug 2017)		Dry season (Feb–Mar 2018)	
	Larvae/habitat	Pupae/habitat	Larvae/habitat	Pupae/habitat
Habitat type				
Buckets	0.12 ± 0.91	0.11 ± 1.41	0.02 ± 0.23	0.01 ± 0.13
Drums	16.97 ± 39.72	3.25 ± 9.22	6.83 ± 25.47	0.92 ± 4.35
Jerrycans	1.71 ± 13.32	0.40 ± 3.10	0.06 ± 0.37	0.01 ± 0.15
Others	-	-	-	-
Pots	2.69 ± 7.61	0.55 ± 2.35	1.51 ± 5.66	0.28 ± 1.63
SDC	0.42 ± 2.79	0.33 ± 3.40	-	-
Tires	8.47 ± 19.76	1.28 ± 5.28	0.53 ± 3.04	0.08 ± 0.66
Habitat stability				
Unstable	0.07 ± 1.34	0.02 ± 0.51	0.27 ± 2.18	0.04 ± 0.79
Stable	8.49 ± 24.25	1.62 ± 6.27	4.78 ± 20.22	0.67 ± 3.57
Place				
Backyard	4.56 ± 19.11	0.88 ± 4.89	1.33 ± 10.84	0.18 ± 1.84
Frontyard	4.87 ± 12.32	0.87 ± 3.17	1.57 ± 5.68	0.32 ± 1.72
Habitat size				
Large	15.39 ± 34.46	2.67 ± 7.99	4.13 ± 19.99	0.57 ± 3.42
Small	2.04 ± 9.92	0.46 ± 3.32	0.48 ± 3.07	0.07 ± 0.77
Habitat purpose				
Domestic chores	3.26 ± 18.09	0.73 ± 4.58	1.82 ± 13.43	0.25 + 2.27
No purpose	7.01 ± 18.44	1.14 ± 4.84	0.80 ± 3.95	0.14 ± 1.03
Habitat filled				
≤Week	0.19 ± 2.09	0.07 ± 1.59	0.06 ± 0.96	0 ± 0
≥Week	2516 ± 36.92	4.67 ± 9.74	9.35 ± 26.05	1.40 ± 4.70
Habitat shade				
Partial shade	4.53 ± 18.90	0.89 ± 4.89	1.56 ± 11.03	0.22 ± 1.94
Full shade	5.1 2 ± 13.58	0.80 ± 2.94	0.08 ± 0.63	0.04 ± 0.58
Cover				
No cover	4.52 ± 18.43	0.94 ± 4.89	1.57 ± 11.13	0.22 ± 1.97
Covered	5.42 ± 17.11	0.30 ± 1.80	0.21 ± 2.11	0.05 ± 0.45
Water source				
Others	1.97 ± 9.05	0.19 ± 1.29	0.10 ± 1.57	0.004 ± 0.11
Rain	7.21 ± 23.91	1.56 ± 6.40	5.88 ± 21.26	0.88 ± 3.82
Move				
No	0	0	0	0
Yes	5.83 ± 20.42	1.12 ± 5.24	1.57 ± 11.05	0.23 ± 1.96

**Fig. 1:**
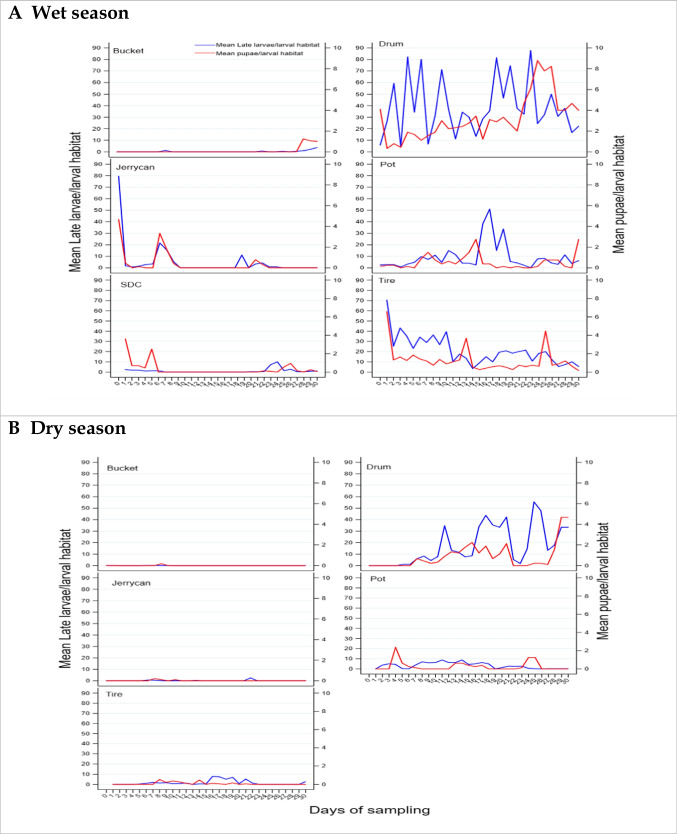
Mean *Ae. aegypti* larvae and pupae per day in different larval habitat types in Bomani town, Kwale County, Kenya

The mean number of larvae and pupae per day in different larval habitat types during the wet and dry season surveys is shown in Fig. 1. Larvae and pupae were distributed at a range of densities across all habitat types (Fig. 1). Mean daily pupal production in all the habitat types for wet and dry are shown in Fig. 1. The mean number of larvae and pupae during wet and dry surveys is presented in Table 5. These data show that habitats typically produced pupae for only a few days within the 30-day sampling period, if they produced pupae at all (Fig. 1; Tables 4 and 5). The predictors for *Ae. aegypti* pupal productivity are shown for wet season in Table 6 and for dry season in Table 7. In the wet season, habitat type was significantly associated with pupal productivity from the multivariate ZINB models (Table 6), with drums, tires, and pots being the key habitat types accounting for 85% (1740/2064) of all pupae sampled. In the dry season, habitat type was significantly associated with pupal productivity in the univariate ZINB models, but multivariate ZINB models did not converge. The key habitats during the dry season were drums and pots accounting for 87% (2207/2537) of all pupae sampled. Of key larval habitats, drums were consistently the most productive habitat for pupae in both seasons (Fig. 1), followed by pots (Fig. 1). Tires which are almost exclusively a rain-filled habitat type were only important during the wet season. Buckets and jerrycans which are commonly used for domestic chores and refilled frequently were rarely found with pupae (Fig. 1). SDC was comparable to jerrycans and pots (Fig. 1, Table 5), but was only productive during the wet season.

**Table 6:** Univariate and multivariate analysis of predictors for *Ae. aegypti* pupal productivity during wet season

	Univariate analysis		Multivariate analysis	
	Crude IRR* (95% CI)	*P* value	Adjusted IRR* (95% CI)	*P* value
Habitat type				
Tire	Reference		Reference	
Bucket	2.21 (1.05, 4.65)	0.04	9.35 (4.61, 18.96)	< 0.001
SDC	1.95 (1.35, 2.82)	< 0.001	5.19 (3.39, 7.95)	< 0.001
Drum	2.16 (0.94, 5.00)	0.04	5.06 (2.27, 11.29)	< 0.001
Jerrycan	1.53 (0.81, 2.91)	0.19	1.68 (1.12, 2.50)	0.01
Pots	0.63 (0.32, 1.27)	0.20	1.23 (0.66, 2.28)	0.52
Habitat stability				
Unstable	Reference		‡	
Stable	1.08 (0.43, 2.72)	0.88	‡	
Place				
Backyard	Reference		Reference	
Frontyard	0.55 (0.28, 1.05)	0.07	0.55 (0.35, 0.86)	0.009
Habitat size				
Large	Reference		‡	
Small	0.70 (0.32, 1.50)	0.36	‡	
Habitat purpose				
Domestic chores	Reference		Reference	
No purpose	0.47 (0.24, 0.93)	0.03	2.62 (2.18, 3.14)	< 0.001
Habitat filled				
< Week	Reference		‡	
≥ Week	0.70 (0.26, 1.92)	0.49	‡	
Habitat shade				
Partial	Reference		Reference	
Full shade	0.55 (0.35, 0.87)	0.01	1.35 (0.94, 1.94)	0.10
Cover				
No cover	Reference		‡	
Covered	0.71 (0.32, 1.58)	0.40	‡	
Water source				
Others	Reference		Reference	
Rain	2.54 (1.69, 3.81)	< 0.001	2.33 (1.69, 3.23)	< 0.001

*IRR* incidence rate ratio

^‡^Variables excluded in the multivariate regression model

^*^IRR and *P* values from the zero-inflated negative binomial regression models

**Table 7:** Univariate and multivariate analysis of predictors for *Ae. aegypti* pupal productivity during dry season

	Univariate analysis		Multivariate analysis	
	Crude IRR* (95% CI)	*P* value	Adjusted IRR* (95% CI)	*P* value
Habitat type				
Tire	Reference		‡	
Bucket	0.05 (0.004, 0.51)	0.01	‡	
Drum	5.03 (1.58, 16.1)	0.006	‡	
Jerrycan	0.08 (0.01, 0.82)	0.03	‡	
Pots	1.42 (0.25, 8.05)	0.69	‡	
Habitat stability				
Unstable	Reference		Reference	
Stable	3.19 (1.55, 6.58)	0.002	1.15 (0.19, 6.88)	0.88
Place				
Backyard	Reference		‡	
Frontyard	0.65 (0.23, 1.85)	0.41	‡	
Habitat size				
Large	Reference		Reference	
Small	0.31 (0.13, 0.74)	0.008	0.05 (0.005, 0.56)	0.01
Habitat purpose				
Domestic chores	Reference		Reference	
No purpose	0.32 (0.14, 0.73)	0.007	4.73 (0.65, 34.19)	0.12
Cover				
No cover	Reference		‡	
Covered	0.37 (0.11, 1.32)	0.13	‡	
Water source				
Others	Reference		Reference	
Rain	6.74 (3.90, 11.7)	< 0.001	2.96 (0.50, 17.73)	0.23
People in the household	1.10 (0.93, 1.29)	0.26	‡	

*IRR* incidence rate ratio

^‡^Variables excluded in the multivariate regression model

^*^IRR and *P* values from the zero-inflated negative binomial regression models

During both surveys, only a few of the habitats were persistently found harboring pupae. During the 30-sampling period in the wet season, pupae were collected from 28% (23/83) of the larval habitats. Further, 54% of the pupae were collected from three habitats: a drum, 31% (640/2064); a tire, 13% (259/2064); and another drum, 10% (209/2064). Pupae were present in these three habitats in 27, 25, and 29 days, respectively, during the 30-day sampling period. In the dry season, only 12% (10/83) of the habitats were ever found with pupae during the 30-day sampling period with three habitats accounting for 80% of all the pupae collected. The proportions of the total pupae contributed by the three drums were 35% (127/365), 33% (121/365), and 12% (45/365). Similarly, in the dry season, the habitats that were the top three sources of pupae were also the most persistent habitats; pupae were present in them in 15, 14, and 8 days, respectively, during the 30-day sampling period.

Habitat stability was defined as the number of days a habitat contained water during the 30-day sampling period. Table S1 shows the mean number of days (stability) the different types of habitats were found with water and the mean number pupae found in each habitat type. In ZINB regression analysis, where stability was treated as a dichotomous variable, stable habitats had more pupae per habitat than did unstable habitats during the wet season, but the difference was not significant (Tables 5 and 6). Stable habitats were significantly more productive than unstable habitats in the univariate ZINB regression analysis, but the effect attenuated in the multivariate analysis. Stability varied considerably by habitat types (Table S1). The four habitat types, buckets, jerrycans, pots, and drums that commonly are used for water storage in the study community, were highly stable (Table S1), but only drums and pots were productive (Tables 5, 6, and 7). “Others” and “SDC” habitat types had very poor stability (Table S1).

Tables 6 and 7 show the results of the multivariate ZINB models for the risk factors of pupal productivity. Habitat type, placing of larval habitats in the backyard (IRR = 0.55 (0.35, 0.86)), larval habitats without purpose (IRR = 2.62 (2.18, 3.14)), and rainwater (IRR = 2.33 (1.69, 3.23)) were the important predictors of larval habitat productivity during the wet season. Although the multivariate model for habitat type did converge, habitat type and large size larval habitats (IRR = 0.05 (0.005, 0.56)) were the only important predictors during the dry season.

## Discussion

The longitudinal estimation of larval habitat productivity deployed in this study revealed important insights in breeding dynamics of *Ae. aegypti* unknown previously. The majority of pupal productivity studies for mosquito vectors employ cross-sectional study designs. The assumption during these one-time sampling surveys is that the mere presence of larvae or pupae in potential larval habitat will eventually translate to adult mosquitoes and that production is stable over time. The longitudinal sampling employed in this study debunks this notion. Only a few of the immatures become adults, and the process is dependent on larval habitat characteristics and human behavior. These results reveal that different mosquito productivity risk factors exist for wet and dry seasons. In the wet season, larval habitat type, purpose of the water in the habitat, source of the water, and whether the larval habitat is located at the back or front of the house were important drivers of pupal productivity. In the dry season, container size was the most important factor influencing pupal production.

This study clearly shows that larval habitat type is an important predictor of pupal productivity. Drums which are usually about 200 l in volume and typically used for water storage were the most productive larval habitats in both seasons. The drum size and uses of the water in the drums enhance their suitability as important sources of *Ae. aegypti* mosquitoes (Burkot et al. 2007; Chadee 2004; Maciel-de-Freitas et al. 2007; Ngugi et al. 2017). Pots were ranked as the second most productive habitats after drums. Like drums, pots in the study area are almost exclusively used for water storage in the study community. Drums and pots are the key sources for the vectors because water storage happens year-round. Buckets, jerry cans, and SDC (which are rarely used for water storage, but rather for other domestic uses such as hygiene, cooking, and drinking) were not as productive compared to the drums and pots, which are used predominantly for water storage. Similar findings were reported elsewhere—containers that were “less active” were more productive compared to the more active containers (Hiscox et al. 2013). Unlike in water storage, frequency of water refilling in larval habitats used for domestic uses is high, and more often there is insufficient time for complete larval development. Additionally, in the wet season, community members usually prioritize use of rain-harvested water in the small-sized containers such as buckets, basins, and jerry cans. This behavior results in drums and pots remaining unattended for extended periods of time, thus enabling undisrupted breeding. Similarly, communities will exhaust rain-harvested water in containers in the front yards before moving to the back yard because they are more accessible and convenient. This creates less active larval habitats in the backyards and thus more productive habitats. Our results confirm prior data that use (purpose) of the water in the larval habitats is an important consideration when designing interventions targeting the key larval habitats (Forsyth et al. 2020).

All the larval habitats surveyed in this study were located in the outdoor domestic environment, and their refilling method was influenced by water use. Larval habitats with no immediate use, such as tires, SDC, and “others,” are exclusively refilled through rain. Previous studies identified rain-filling as an important characteristic of *Ae. aegypti* larval habitats; more immatures were observed in rain-filled larval habitats (Forsyth et al. 2020; Hammond et al. 2007; Morrison et al. 2004; Ngugi et al. 2017). Dependency on rainwater for refilling explains why they were largely only productive during the wet season. These findings suggest that SDC and “others” are not as important sources of *Ae. aegypti* mosquitoes as previously reported in cross-sectional studies in the study area (Forsyth et al. 2020; Ngugi et al. 2017). These habitats were observed to be typically small, with very low stability and often with no immediate use. As an important application of these findings, regular targeted community clean-up of these unattended containers and creation of awareness (SDC, “other,” and tires) is an essential component of vector control in the study area. This recommendation follows because there is very little knowledge on breeding and control strategies of *Ae. aegypti* in the study area, but there is adequate knowledge on breeding and control of malaria vectors (Forsyth et al. 2020).

Tires are predominantly located outdoor, typically rain-filled, more stable compared to SDC and “others,” and are less abundant in the study area. Tires usually did have specific uses, and were often left unattended in the outdoor peridomestic area, explaining their relatively high productivity. As a key breeding site in the wet season, efforts are required to ensure they do not collect rainwater. Covering the tires or re-purposing them to make recycled goods such as toys or shoes can help to control ongoing breeding in the tires (Forsyth et al. 2020). Overall, targeted source reduction in the three main “key larval habitats” (drums, pots, and tires) may be augmented by improved yard management (Barrera et al. 2006b).

Large volume larval habitats have been reported consistently as important for immature *Aedes* production (Barrera et al. 2006b, 1993; Islam et al. 2019; Maciel-de-Freitas and Lourenço-de-Oliveira 2011; Morales-Pérez et al. 2017); however, during the wet season when all outdoor habitats have equal opportunity of being refilled through rain, size and stability of the larval habitats become less important. Instead, how “active” or the “habitat purpose” matters more. Because rain events are infrequent during the dry season, large and stable containers become more productive, as they are able to hold water long enough to allow for complete larval development. Identification of “key habitats” for targeted intervention is therefore more feasible during dry season. Understanding the difference in pupal productivity between habitats in the different seasons is important to design effective control strategies over the year.

During the baseline cross-sectional survey, only larval habitat type and the ability of the habitat’s movability were associated with *Ae. aegypti* immatures. While the larval habitat type was identified a key factor influencing larval habitat productivity by longitudinal surveys, larval habitat movability was not. The majority of the movable larval habitats such as “SDC” and “others” were demonstrated as poor sources of *Ae. aegypti* mosquitoes. These findings point to a key weakness of one-time cross-sectional vector surveys. The current method of estimating larval habitat productivity accounted for the temporal dynamics in the breeding ecology of *Ae. aegypti* mosquitoes, a more accurate way of identifying the “key larval habitats.” We propose that the method used here is a better way of estimating the entomological risk associated with different larval habitat types and is recommended when designing *Ae. aegypti* control interventions. Longitudinal surveys are required in different geographical locations, because key larval habitats are known to differ with locality.

In conclusion, drums, pots, and tires were sources of more than 85% of *Ae. aegypti* pupae, reinforcing the key container concept. Targeting these three types of habitats makes epidemiological sense especially during the dry season. All three are relatively large in size and easily identifiable, and thus amenable to source reduction intervention programs. Correctly covering drums and pots may be a practical source reduction control strategy for the study communities in South Coast Kenya. Maintaining cleanliness in the peridomestic area especially involving removal of all potential larval habitats during the wet season when there are numerous and diverse containers that have the potential of becoming larval habitats could be a key strategy. Regular clean-up events could greatly reduce breeding in SDCs and tires. This may however require sustained and continuous awareness creation.

## Supplementary Information

Below is the link to the electronic supplementary material.

Supplementary file1 (DOC 37 KB)

## Data Availability

The datasets used during and/or analyzed during the current study are available from the corresponding author on request.

## Abbreviations

VBDCU: Vector-Borne Disease Control Unit
SDC: Small domestic containers
AFC: Animal feeding containers
ZINB: Zero-inflated negative binominal regression
IRR: Incidence rate ratios
