# Supplementary material for: Pupal productivity of larval habitats of Aedes aegypti in Msambweni, Kwale County, Kenya
Source: Parasitol Res. 2023 Jan 23;122(3):801–14. doi: 10.1007/s00436-022-07777-0 (PMC9988718; doi:10.1007/s00436-022-07777-0)
Supplement: Supplementary file 1 — Supplementary file1 (DOC 37 KB) [file 436_2022_7777_MOESM1_ESM.doc]

**Supplementary Material**

Table S1: Stability of the larval habitats during the 30-day sampling period

| **Habitat type** | **Number of larval habitats** | **Wet season stability**  **(95% CI)** | **Mean pupae/larval habitat** | **Dry season stability**  **(95% CI)** | **Mean pupae/larval habitat** |
| --- | --- | --- | --- | --- | --- |
| Buckets | 17 | 12.52 (7.28-17.77) | 3.29 | 13.88 (9.18-18-58) | 0.18 |
| Drums | 9 | 16.55 (8.10-25.01) | 97.44 | 15.44 (4.34-26.54) | 27.56 |
| Jerrycan | 9 | 14.89 (8.24-21.54) | 11.89 | 14.88 (6.66-23.12) | 0.44 |
| Others | 5 | 5.80 (-4.68-16.28) | 0.00 | 0.40 (-0.71-1.51) | 0.00 |
| Pots | 8 | 12.50 (0.99-24.01) | 16.63 | 13.00 (2.88-23.12) | 8.25 |
| SDC | 16 | 3.81 (0.29-7.33) | 10.06 | 1.25 (-0.19-2.44) | 0.00 |
| Tires | 19 | 13.00 (7.83-18.17) | 38.42 | 8.84 (4.33-13.35) | 2.32 |
| **Total** | **83** | **11.24 (8.94-13.54)** | **24.87** | **9.65 (7.34-11.96)** | **4.40** |
